# Different Outcomes of Hypospadias Surgery Between North America, Europe and China: Is Patient Age a Factor?

**DOI:** 10.5812/numonthly.1853

**Published:** 2012-09-24

**Authors:** Wenli Lu, Yuehong Tao, Amy B. Wisniewski, Dominic Frimberger, Brad P. Kropp

**Affiliations:** 1 Department of Pediatrics, Shanghai Ruijin Hospital, Medical School of Shanghai Jiao Tong University, Shanghai, China; 2 Department of Pediatrics, Subei People’s Hospital of Jiangsu Province, Jiangsu, China; 3 Department of Urology, University of Oklahoma Health Sciences Center, Oklahoma City, USA

**Keywords:** Hypospadias, General Surgery, Outcomes Assessment

## Abstract

**Background and Objects:**

The patient’s age at the first hypospadias repair may be an important factor for determining postoperative outcomes. Age at the first procedure differs between Western countries and medical centers in China. This review examines the differences between the incidence of surgical complications and surgical age in boys receiving hypospadias repairs in North America, Europe and China.

**Materials and Methods:**

Literature reports were reviewed in PubMed and WanFang databases using the key terms and phrases; ‘hypospadias outcomes’, ‘complications of hypospadias repair’ and ‘timing of hypospadias repair’. All peer-reviewed articles published over the past decade (2001-2011) were considered if; a full text was available, the article included age at the first hypospadias procedure and surgical complications.

**Results:**

In total, 16 131 patients were reported in 113 papers from North America, Europe and China according to our inclusion criteria. There was a significant difference in age at the first hypospadias surgery (P < 0.0005) and in the incidence of complications (P <0.001) between the different global regions investigated, with the earliest surgeries occurring in North American patients. Urethral fistulas were the most common complication reported in all of the regions included in this study.

**Conclusions:**

Fellowship training in pediatric urology could improve surgical outcomes, particularly in young children. Younger children experience fewer complications following hypospadias surgeries, independent of training and access to resources.

## 1. Introduction

Hypospadias, is a congenital malformation of the male urethra, it is a common birth defect in humans and is purported to be increasing in incidence (1). Variables affecting short- and long-term outcomes of hypospadias repair include, but are not limited to; the degree of malformation present, type of surgical procedure employed for the repair, and the training and experience of the surgeons performing the repair. Current recommendations from pediatric medical societies in the United States and Europe are to perform hypospadias surgery between 6 and 12 months of age (2, 3). In countries such as China hypospadias repair usually occurs in older boys, typically between 4 to 6 years of age, as most surgeons in China do not practice in specialized pediatric facilities nor do they complete pediatric sub-specialty training. This difference in surgical approach is of potential concern, because older patients who receive hypospadias surgery, are more likely to report delayed sexual activity and sexual dissatisfaction (4).

The aim of the current review is to compare the age at first surgery for hypospadias repair and surgical complications (type and incidence) from medical research databases that include peer-reviewed, outcome studies written in English (Pubmed) and Chinese (WanFang). The inclusion of the WanFang database allows for a direct comparison of differences in surgical practice and outcomes between surgeons who train and practice in Western countries versus those in China. Of interest is whether or not a later age at the first surgical procedure is associated with more complications and/or unique types of post-surgical complications following hypospadias repair.

## 2. Materials and Methods

Hypospadias outcome studies from 2001 through 2011 were identified using the Pubmed and WanFang databases. Search terms and phrases included; ‘hypospadias outcomes’, ‘complications of hypospadias repair’ and ‘timing of hypospadias repair’. Studies that reported the age of patients at the first surgery, degree of hypospadias and type (s) of procedure used were reviewed. When comparing across global regions, for differences in age, complication type and incidence, only first time surgeries were considered.

## 3. Results

A total of 113 studies were selected from North America (n = 30), Europe (n = 30) and China (n = 53; 19 published in English and 34 published in Chinese; see Table 1). When combined, a total of 16 131 patients in these studies received surgical repair for hypospadias. More than 6 000 patients presented with distal hypospadias, typically requiring a one-stage procedure to repair. Others presented with a more severe degree of hypospadias that in some cases required a two- or three-stage procedure to repair (see Table 2). One-stage procedures typically included urethroplasty using free inner prepuce or bladder mucosa, TIP, MAGPI, Mathieu, Duckett and Duplay procedures.

**Table 1:** Description of Papers and Patients Reviewed

	Paper (n)	Patients (n)	Age at First Surgery (mo) (Mean ± SEM)	Complications No. (%)
**North America**	30	3 797	20.60 ± 2.49	412 (10.9)
**Europe**	30	3 392	31.70 ± 2.85	540 (15.9)
**China**	53	8 942	67.14 ± 4.87	1 332 (14.9)
**Total**	113	16 131		2284

**Table 2:** Type and Stages of Hypospadias Surgery

	Distal	Stage	No.	Proximal	Stage	No.	Severe	Stage	No.
**North America, n**	2076			1230			228		
		1-stage	2073		1-stage	978		1-stage	102
		Not reported	3		2-stage	208		2-stage	126
					Not reported	44		Not reported	0
**Europe, n**	2967			211			219		
		1-stage	293		1-stage	69		1-stage	93
		Not reported	31		2-stage	122		2-stage	121
					Not reported	20		Not reported	5
**China, n**	1273			2327			1656		
		1-stage	1165		1-stage	1837		1-stage	1089
		Not reported	108		2-stage	345		2-stage	245
					Not reported	145		Not reported	322

As expected, there was a difference in age at the first hypospadias surgery between the different global regions investigated (F (2) = 38.00, P < 0.0005). North America hypospadias patients typically received their first procedure during the first two years, European patients during the first three years, and Chinese patients during the first five years of life. Many studies reported postoperative complications such as; urethral fistulas, urethral strictures and glans dehiscence (see Table 3). The lowest complication rates were reported by North American investigators and the highest rates occurred among surgeons practicing in China (X^2^ (2) = 46.3, P < 0.001). Urethral fistulas were the most common complication reported for all regions, but they were most commonly reported by surgeons in China (X^2^ (3) = 68.509, P < 0.005; see Table 3).

**Table 3:** Number and Type of Post-operative Complications by Region.

		n	Fistula No. (%)	Stricture No. (%)	Glans Dehiscence No. (%)
**North American**					
	Complication	412	194 (5.1)	17 (0.4)	24 (0.6)
	No complication	3385			
**Europe**					
	Complication	540	257 (7.6)	34 (1.0)	66 (1.9)
	No complication	2852			
**China**					
	Complication	1332	843 (9.4)	15 (0.2)	312 (3.5)
	No complication	7610			

## 4. Discussion

In regions of the world where specialized pediatric surgical training exists, age at the first hypospadias procedure occurs in younger patient and is associated with lower complication rates (5-7). This is in line with recommendations for early surgery, prior to 18 months of life or after three years if an earlier opportunity is not possible (3). A possible explanation for the lower complication rates reported by studies from North America and Europe is the difference in training and facilities geared toward pediatric surgery compared with China (5). Specifically, fellowship training in pediatric urology improves surgical outcomes, particularly in young children (6, 7). Thus, subspecialty training in pediatric surgery most likely contributes to some of the regional differences reported here. Establishing pediatric surgical training programs in China, similar to those in Europe and North America, would be expected to result in reduced complication rates following hypospadias repairs in these patients.

A second potential explanation for our results is that younger children heal better following surgical procedures, regardless of the surgeon’s training and resources. In studies that considered age at surgery within institutions that controlled for surgeon training and experience level, complications were minimized when hypospadias repair was performed in younger patients (8-11).

In summary, early hypospadias surgery may be associated with better long-term outcomes (2, 4). Additionally, an inverse relationship exists between age at surgery and memory of the operation, therefore patients who do not remember the earlier procedures, report greater satisfaction with the cosmetic outcome (12). Performing surgeries for hypospadias repair at later ages may predispose patients to a greater likelihood of post-surgical complications, the most common being urethral fistulas. The development of pediatric urology training programs in China may ultimately lead to better outcomes of hypospadias procedures, this could be due in part to greater levels of comfort in treating younger aged patients.
